# Mixed Matrix Membranes for O_2_/N_2_ Separation: The Influence of Temperature

**DOI:** 10.3390/membranes6020028

**Published:** 2016-05-16

**Authors:** Ana Fernández-Barquín, Clara Casado-Coterillo, Susana Valencia, Angel Irabien

**Affiliations:** 1 Department of Chemical and Biomolecular Engineering, Universidad de Cantabria, Av. Los Castros s/n, Santander 39005, Spain; fbarquina@unican.es (A.F.-B.); irabienj@unican.es (A.I.); 2 Instituto de Tecnología Química, Universitat Politècnica de València—Consejo Superior de Investigaciones Científicas, Av. de Los Naranjos s/n, Valencia 46022, Spain; svalenci@itq.upv.es

**Keywords:** zeolites, Si/Al = 5, poly(trimethylsilylpropyne) (PTMSP), Rho, chabazite, LTA, temperature, oxygen, nitrogen

## Abstract

In this work, mixed matrix membranes (MMMs) composed of small-pore zeolites with various topologies (CHA (Si/Al = 5), LTA (Si/Al = 1 and 5), and Rho (Si/Al = 5)) as dispersed phase, and the hugely permeable poly(1-trimethylsilyl-1-propyne) (PTMSP) as continuous phase, have been synthesized via solution casting, in order to obtain membranes that could be attractive for oxygen-enriched air production. The O_2_/N_2_ gas separation performance of the MMMs has been analyzed in terms of permeability, diffusivity, and solubility in the temperature range of 298–333 K. The higher the temperature of the oxygen-enriched stream, the lower the energy required for the combustion process. The effect of temperature on the gas permeability, diffusivity, and solubility of these MMMs is described in terms of the Arrhenius and Van’t Hoff relationships with acceptable accuracy. Moreover, the O_2_/N_2_ permselectivity of the MMMs increases with temperature, the O_2_/N_2_ selectivities being considerably higher than those of the pure PTMSP. In consequence, most of the MMMs prepared in this work exceeded the Robeson’s upper bound for the O_2_/N_2_ gas pair in the temperature range under study, with not much decrease in the O_2_ permeabilities, reaching O_2_/N_2_ selectivities of up to 8.43 and O_2_ permeabilities up to 4,800 Barrer at 333 K.

## 1. Introduction

Oxygen production from air separation can be used in many chemical and environmental applications such as medical devices, steel, and chemical manufacturing as well as in the combustion enhancement of natural gas and coal gasification [1]. Cryogenic distillation and pressure swing adsorption (PSA) are the current technologies for oxygen-enriched air production. However, these techniques are still considered as too energy intensive. Less energy-intensive air separation methods are critically necessary for emerging applications such as oxy-combustion carbon capture, furnaces, and reformers [2]. If oxygen-enriched air (OEA) is used during fuel combustion, the energy loss produced by the dilution of the nitrogen of the air is reduced. Consequently, the efficiency of the heating system and the productivity increases. Besides, the higher the temperature of the oxygen-enriched stream, the lower the energy needed to carry out the combustion [3].

Membrane technology is an attractive alternative in terms of modular design, ease of scaling up and controlling, and low energy requirements. For O_2_/N_2_ gas separation, only polymeric and ion transport membranes (ITMs) have been studied. ITMs have exceptional high selectivities. However, ITMs present difficulties in proper sealing, as well as high sensitivity of membranes to temperature gradients, which sometimes results in membrane cracking [4]. Polymeric membrane materials provide a wide range of permeability and selectivity combinations. However, for gas separation applications, only the most permeable polymers with a particular selectivity with comparable mechanical and thermal properties in the long term could be a potential alternative to inorganic membranes [5]. Nowadays, commercially available polymeric membranes are not able to economically produce O_2_ purity on a large scale compared to conventional techniques, due to the fact that most commercial gas separation membranes are based on low permeable polymers with large selectivity, which increases costs and space requirements [6]. This is, in part, due to the lack of high-temperature stability or insufficient performance in reference to selectivity and flux, where polymeric membranes usually undergo a trade-off limitation between selectivity and permeability, which was well described by Robeson in his upper bound [7].

Development of membrane materials with enhanced properties on the production of OEA is an area of continuous research, due to the potential of oxygen-enriched gas streams in combustion processes for OEA gas production processes [8]. Koros and Mahajan reported that membranes should present a selectivity from 4 to 6 and an oxygen permeability higher than 250 Barrer to be attractive in oxygen separation processes [4]. The highest O_2_/N_2_ selectivities found in polymers in the literature are obtained by glassy polyimides such as PES (poly ether sulfone) [9], PVAc (poly vinyl acetate) [10], Matrimid [11], or 6FDA-TAB (4,4′-(Hexafluoroisopropylidene)diphthalic anhydride-1,2,4,5-tetraaminobenzene) polypyrrolone copolymer [12,13], with O_2_/N_2_ values of up to 7. However, all of them reach very low O_2_ permeabilities; thus, upgrading the membrane materials by improving both permeability and selectivity is a critical issue in order to make the membrane process more competitive and energy-efficient [2].

Mixed matrix membranes (MMMs) are a well-known means by which the properties of polymeric membranes are enhanced. Their microstructure allows the synergistic combination of the polymer’s processing feasibility and the molecular sieving effect and the superior gas separation performance of the inorganics, achieving a hybrid material with improved functional and mechanical properties [14,15]. Materials selection is a key aspect in the successful development of MMMs. The proper selection of the polymeric matrix and the dispersed fillers, as well as a good interaction between the two phases, the control of the filler concentration, shape, and dimensions are important factors to reach the expected performance with a defect-free MMM with synergistic properties [11,16]. Zeolites were the first fillers proposed in MMMs for gas separations due to their molecular sieving capacity, crystalline nature, well-delimited pore structures, and shape separation characteristics. Moreover, small pore zeolites are able to discriminate between molecules with kinetic diameters similar to O_2_ and N_2_ [17]. Although there are already databases on the effect of temperature on polymer films [18,19], there is still work to do on MMMs.

Poly (1-trimethylsilyl-1propyne) (PTMSP) has been proposed in previous works [20,21] as the continuous polymeric matrix in the CO_2_/N_2_ gas pair separation due to its high permeability, which determines the minimum separation performance. Small-pore zeolites with different frameworks (CHA, LTA and Rho), Si/Al ratios (1, 5 and ∞), and zeolite loadings (5, 10 and 20 wt %) were selected as fillers. The MMMs that showed the best separation ability were the 5 wt % CHA-PTMSP, the 5 wt % LTA5-PTMSP, the 5 wt % Rho-PTMSP, and the 20 wt % LTA1-PTMSP, with a slight decrease in permeability but a considerable enhancement in selectivity, surpassing the Robeson’s upper bound, even increasing the temperature up to 333 K. The O_2_ permeability of PTMSP reported in the literature covers a range between 6000 and 10,000 Barrer at 308 K, decreasing with temperature. However, its high permeability is accompanied with a very low O_2_/N_2_ selectivity, with values between 1.30 and 1.70 at 308 K [22,23,24,25]. Consequently, the incorporation of suitable chosen inorganic fillers is likely to improve the selectivity of a defect-free membrane [17,26,27,28,29].

Accordingly, in this work, the membrane materials that presented the best CO_2_/N_2_ gas separation permselectivity have also been selected to study their performance in the O_2_/N_2_ separation. These membrane materials consist of MMMs prepared from the highly permeable glassy PTMSP polymer and small-pore chabazite (Si/Al = 5), Rho (Si/Al = 5), and LTA (Si/Al = 1 and 5) zeolites. We studied the effect of these fillers, with different topologies, in pure O_2_ and N_2_ gas permeability tests under the same temperature range as in previous studies, namely, 298–333 K.

## 2. Results

The thermal resistance of the MMMs is as high as that of pristine PTMSP membranes, up to 573 K, which accounts for the potential use of these MMMs at industrial levels where separation processes are carried out at elevated temperatures [21]. The real zeolite loading of the MMMs was determined from the residual weight after TGA experiments, as reported in a previous work [21]. In all cases, the real zeolite loading agreed with nominal values, being 7.58 ± 3.30 wt %, 8.48 ± 3.55 wt %, 7.65 ± 2.72 wt %, and 28.6 ± 8.84 wt % for 5 wt % CHA-PTMSP, 5 wt % LTA5-PTMSP, 5 wt % Rho-PTMSP, and 20 wt % LTA1-PTMSP MMMs, respectively.

**Figure 1:**
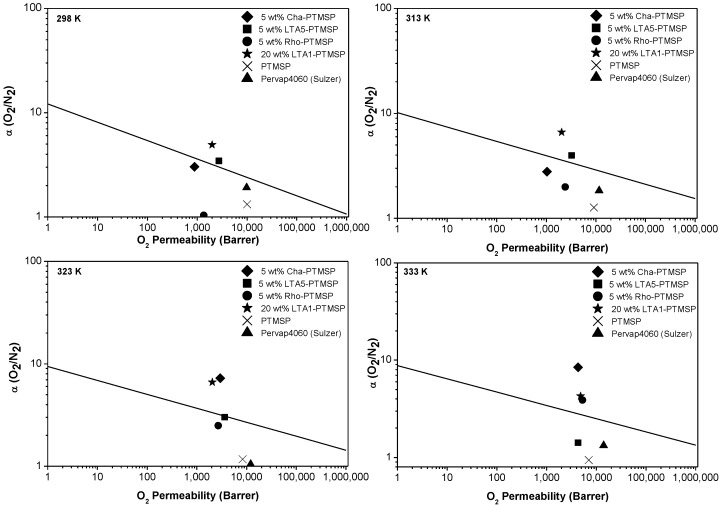
Permeability and selectivity values of the MMMs in this work, in comparison with the Robeson’s upper bound for O_2_/N_2_ separation at 298 K, 313 K, 323 K, and 333 K.

The O_2_/N_2_ permselectivity values of the MMMs as well as those of the pure PTMSP membranes studied in this work are included in Figure 1, compared with Robeson’s upper bound at different temperatures. The upper bound vertical shifts with temperature have been calculated taking into account the parameters calculated by Rowe *et al.* [30], according to whom the upper bound for the gas pair under study moves downwards with increasing temperature. The permselectivities of all the MMMs in this work are enhanced with rising temperature, with the exception of 5 wt % LTA5-PTMSP MMMs. This was also observed in the CO_2_/N_2_ gas pair separation in the same temperature range [21]. At 298 K and 313 K, Robeson’s upper bound is surpassed by the 5 wt % LTA5-PTMSP MMM and the 20 wt % LTA1-PTMSP MMM—at 323 K, by the 5 wt % LTA5-PTMSP MMM, the 5 wt % CHA-PTMSP MMM, and the 20 wt % LTA1-PTMSP MMM. At 333 K, the upper bound is surpassed by the 5 wt % CHA-PTMSP MMM, the 5 wt % Rho-PTMSP MMM, and the 20 wt % LTA1-PTMSP MMM. In all cases, the selectivity is much higher than that of the pure PTMSP membranes tested in the laboratory and that of the commercial membrane Pervap 4060 (Sulzer Chemtech, Alschwill, Switzerland) used for comparison purposes. Thus, the molecular sieving effect of the small-pore zeolites to the polymer matrix and the absence of defects due to good compatibility between the fillers and the polymer matrix [20,21] contributes to overcoming the separation performance of other MMMs reported in the literature [9,10,11,31].

The influence of temperature in the O_2_/N_2_ gas separation performance of these MMMs has been analyzed in terms of gas permeability and gas diffusivity in the range from 298 to 333 K. The O_2_ permeability values as well as the O_2_/N_2_ selectivities of the different MMMs studied in this work are collected in Table 1, as well as those of other MMMs reported in the literature. The O_2_ diffusivities and O_2_/N_2_ diffusivity selectivities are presented in Table 2, whereas the O_2_ solubilities and O_2_/N_2_ solubility selectivities calculated by Equation (6) are shown in Table 3, also as a function of temperature.

Both the O_2_ permeability and diffusivity through the pure PTMSP membranes decrease with temperature. O_2_ permeability decreases from 10,083 ± 936 Barrer at 298 K to 7024 ± 1050 Barrer at 333 K, while O_2_ diffusivity diminishes from 5.47 × 10^−8^ ± 1.36 × 10^−8^ cm^2^·s^−1^ at 298 K to 3.46 × 10^−8^ ± 7.72 × 10^−9^ cm^2^·s^−1^ at 333 K. These values are in good agreement with those reported in the literature [17,22,23,24,25,32,33]. However, in spite of the high oxygen permeability, the ideal O_2_/N_2_ selectivity of pure PTMSP is very low, ranging from 1.32 ± 0.12 at 298 K to 0.94 ± 0.41 at 333 K. This is a characteristic behavior of PTMSP due to its high free volume and the fact that the weakly and rigid molecular sieving framework is more prone to changes in solubility than diffusivity [24,32].

On the contrary, the permeability of the MMMs prepared in this work increases with temperature, and the O_2_/N_2_ selectivity is considerably enhanced compared to that of the pure polymer in the temperature range under study. This is attributed to the molecular sieving effect imparted by the introduction of the zeolites, as well as the good interaction between the inorganic fillers and the polymer matrix, which leads to a simultaneous effect of adsorption and surface diffusion properties in the MMMs that were not present in the pure PTMSP membrane [34]. The O_2_ permeability of the 5 wt % CHA-PTMSP MMM rises from 885 to 4308 Barrer, from 2727 to 4316 Barrer for the 5 wt % LTA5-PTMSP MMM, from 1368 to 5312 Barrer for the 5 wt % Rho-PTMSP MMM, and from 2000 to 4833 Barrer for the 20 wt % LTA1-PTMSP MMM when rising the temperature from 298 to 333 K. At the same time, it can be seen from Table 1 that the O_2_/N_2_ selectivity of the 5 wt % CHA-PTMSP MMM increases from 3.03 to 8.43, whereas the 5 wt % Rho-PTMSP only from 1.03 to 3.88, and the 5 wt % LTA5-PTMSP and the 20 wt % LTA1-PTMSP selectivities maintain almost constant in temperature, with values around 3.50 and 5, respectively. The O_2_/N_2_ ideal selectivity of the MMMs in the entire temperature range under study is higher than that of the pristine PTMSP polymer membrane measured under the same conditions. That is the reason why, in spite of the fact that the O_2_ permeabilities decrease compared to that of the pure polymer membrane, the permselectivity obtained with the MMMs is higher than that of the pure PTMSP membrane, especially at high temperatures. It can also be observed that the most selective cases are those with the highest diffusion and sorption values (Table 2 and Table 3). Noteworthy, MMMs that give the highest permeabilities are those with the largest pore-size zeolites (Table 1). This improves the diffusivity contribution to permeation compared with others [21,35]. The slight O_2_ permeability reduction in the MMMs compared to that of the pristine polymer could be attributed to different phenomena such as chain rigidification of the polymer near the surface of the zeolite particles, which reduces the penetration of gases on that region, or partial blockage of the zeolite pores by the polymer chains [36].

In the same way, it can be appreciated from Table 2 and Table 3 that O_2_ diffusion and sorption coefficients are higher than those of N_2_, combining the contribution of diffusivity and solubility in permeability due to the solution–diffusion model. Moreover, in general, the diffusion coefficients increased with higher zeolite loading, those of 20 wt % LTA1-PTMSP being the highest. The presence of zeolite fillers increases the diffusion time lag because of immobilizing adsorption sites so that additional time is required to accumulate the excess penetrant before a steady state can be reached, thus resulting in a diffusivity decrease [31]. The diffusion coefficients of gases increased with decreasing filler particle size; diffusivity O_2_ (5 wt % LTA5-PTMSP) > diffusivity O_2_ (5 wt % CHA-PTMSP) > diffusivity O_2_ (5 wt % Rho-PTMSP), in agreement with the literature [37].

**Figure 2:**
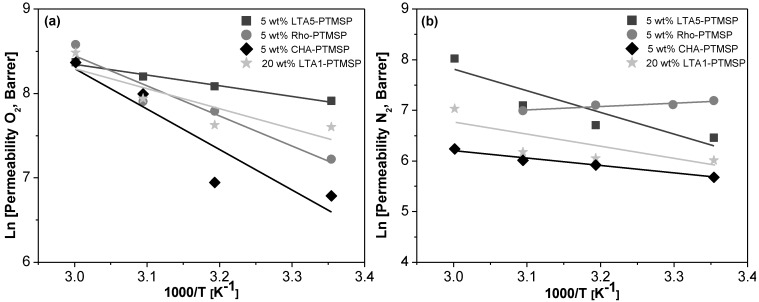
Arrhenius plots of the O_2_ (**a**) and N_2_ (**b**) permeability *vs.* the reciprocal of temperature.

**Figure 3:**
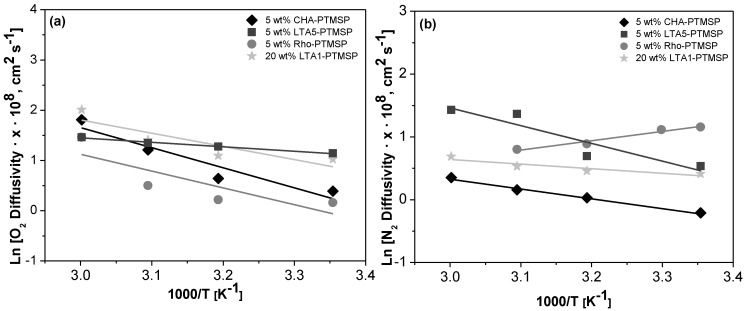
Arrhenius plots of the O_2_ (**a**) and N_2_ (**b**) diffusivity *vs.* the reciprocal of temperature.

**Figure 4:**
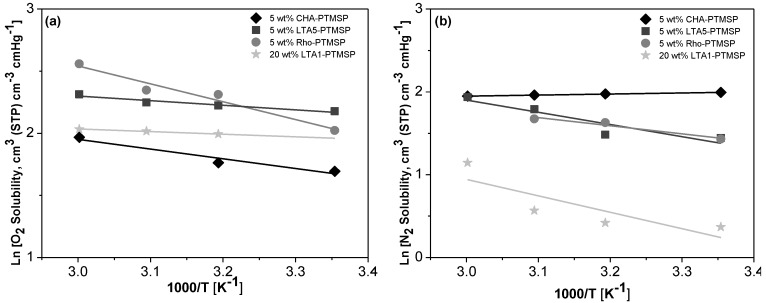
Van’t Hoff plots of the O_2_ (**a**) and N_2_ (**b**) solubility *vs.* the reciprocal of temperature.

Arrhenius and Van’t Hoff plots are presented in Figure 2, Figure 3 and Figure 4, respectively. A good correlation (R^2^ = 0.90 ± 0.08) is observed in all cases. Therefore, the effect of temperature on the gas permeability and diffusivity is well described in terms of Arrhenius and Van’t Hoff relationships by Equations (3)–(5). The activation energies for permeation are −8.19 ± 1.72 kJ·mol^−1^ and −12.60 ± 4.10 kJ·mol^−1^ for O_2_ and N_2_, respectively, through pure PTMSP membranes, in agreement with the literature [23,24]. In this work, the activation energies for diffusion are −11.02 ± 1.71 kJ·mol^−1^ and −17.29 ± 8.34 kJ·mol^−1^ for O_2_ and N_2_, respectively, whereas the activation energies for sorption are −2.34 ± 1.89 kJ·mol^−1^ and 5.36 ± 2.32 kJ·mol^−1^ for O_2_ and N_2_, respectively. The higher the temperature effect on the permeation, diffusion or sorption rate is, the higher is its energy activation value [18]. The different behavior observed of N_2_ permeability and diffusivity with temperature in the 5 wt % Rho/PTMSP MMM could be attributed to the accumulation of the zeolite particles at the bottom of the membrane, forming clusters of agglomerates as observed by SEM [21]. Regarding the lower N_2_ solubility of the 20 wt % LTA1-PTMSP MMM compared to the others, this can be related to the high O_2_/N_2_ solubility selectivity compared to others, which has a greater contribution than diffusivity in permeability [19].

The activation energies of permeation, diffusion, and sorption through the MMMs are presented in Table 4. In pure PTMSP membranes, the activation energy of permeation, diffusion, and sorption are negative, as reported by other researchers. In the case of almost all the MMMs studied in this work, the activation energies present positive values. The rise of activation energies for the permeation indicates that an interaction phenomenon occurs when the zeolites are added to the PTMSP matrix to form the MMM. This reveals an interaction between the dispersed fillers and the PTMSP matrix [20,21,23]. The fact that the activation energies of permeation of the MMMs are higher than those of the pristine PTMSP reveals again that the adsorption capacities of the zeolites are also taking part in the transport mechanism, as observed in the influence on the solubility of the MMMs collected in Table 3 [21].

The permeability dependency is a combination of the diffusion and solubility coefficients temperature dependencies. These two parameters show competing effects; however, in this work, in general, the activation energy of permeation is, practically, the addition of the activation energies of diffusion and sorption. The higher the temperature effect on the diffusion or sorption rate, the higher the energy activation for the diffusion or sorption, and the higher the influence of diffusivity or solubility on permeation [38].

Since the pore size of the zeolites employed as fillers are 3.6, 3.8, 4, and 4 Å for Rho, CHA, LTA5, and LTA1, respectively—very similar to the kinetic diameter of O_2_ and N_2_, which is 3.46 and 3.64 Å, respectively—the zeolite fillers have a significant effect on the diffusion rates of oxygen and nitrogen [39].

**Figure 5:**
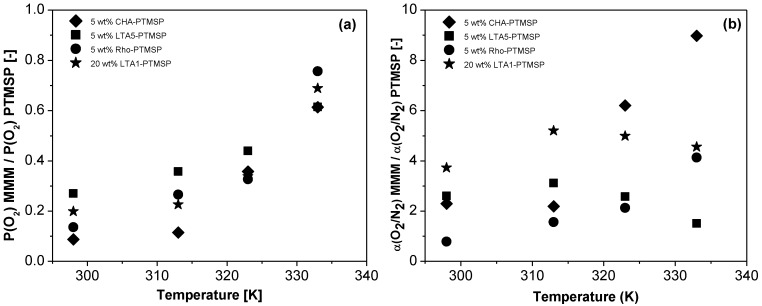
Relative O_2_ permeability (**a**) and relative O_2_/N_2_ selectivity (**b**) of the different MMMs with respect to that of pure PTMSP membranes *versus* temperature.

The relative O_2_ permeability and the relative O_2_/N_2_ selectivity of the different MMMs with respect to that of the pure PTMSP membranes are plotted *versus* temperature in Figure 5a,b, respectively. The permeabilities of the MMMs are lower than those of the pure membranes. However, in all cases, they increase with temperature. The O_2_ permeability of the 5 wt % CHA-PTMSP MMM increases from 0.09 times that of the pure PTMSP membrane at 298 K to 0.61 times at 333 K. The rest of the MMMs behave in a similar manner, rising from 0.27 times, 0.14 times, and 0.20 times at 298 K to 0.61 times, 0.76 times, and 0.69 times those of the pure PTMSP for the 5 wt % LTA5-PTMSP, 5 wt % Rho-PTMSP and 20 wt % LTA1-PTMSP MMMs at 333 K, respectively. Regarding the relative O_2_/N_2_ selectivities with respect to those of PTMSP, it is worth noting that all of them are higher than 1.0, *i.e.*, all are much higher than those of the pristine polymer membrane. The selectivity of the 5 wt % CHA-PTMSP and 5 wt % Rho-PTMSP MMMs reach values up to 8.98 or 4.13 times higher than that of the PTMSP membrane at 333 K, respectively. The O_2_/N_2_ selectivity of the 5 wt % LTA5-PTMSP and 20 wt % LTA1-PTMSP membranes are 3.12 times greater than that of pure PTMSP membranes at 313 K.

## 3. Materials and Methods

Poly(1-trimethylsilyl-1-propyne) polymer (PTMSP, ABCR GmbH, Karlsruhe, Germany), with a purity of 95%, was used after being dried at 343 K for one hour to eliminate possible humidity and impurities. Toluene was purchased from Sigma-Aldrich (Toluene, anhydrous, 99.8%, Sigma-Aldrich Quimica S.L., Barcelona, Spain).

Zeolites, LTA5 (Si/Al = 5), Chabazite (Si/Al = 5), and Rho (Si/Al = 5), were synthesized in the Instituto de Tecnología Química in Valencia, and zeolite LTA1 (Si/Al = 1) (molecular sieves 4 A) was purchased from Sigma-Aldrich. The particle size of the zeolites is 0.5 µm (LTA5), 1.0 µm (CHA), 1.5 µm (Rho), and 2.5 µm (LTA1) [21].

### 3.1. Synthesis of MMM

In this work, MMMs were prepared by the solution casting method, following the procedure described in our previous works [20,21]. In a typical synthesis, 1.5 wt % PTMSP was dissolved in toluene, and the PTMSP solution was then filtered under vacuum to eliminate insoluble impurities. At this point, the zeolites, previously dispersed in the solvent for 2 h, were added to the polymer solution and stirred for other 24 h. Then, 10 mL of dissolution were degassed in an ultrasound bath and cast on a glass plate. Membranes were dried slowly to a constant weight, covered by a Petri dish at ambient conditions, to ensure a slow evaporation of the solvent. This technique ensures the absence of mechanical damage or contraction of the synthesized membrane. The membranes are stored in the laboratory and quickly immersed in methanol before the permeation tests to avoid aging and assure a reproducible and reliable permeation performance.

The membrane thickness was measured by means of a digital micrometer (Mitutoyo digimatic micrometer, IP 65, Mitutoyo, Kawasaki, Japan) with an accuracy of 0.001 mm. The average thickness of the membranes is 72.92 ± 4.97 μm, not being affected by the zeolite topology. As the membranes present similar thickness, the differences in permeability of the MMMs are not attributed to the effect of this parameter.

### 3.2. Characterization

The synthesis and characterization of the MMMs studied in this work was reported in previous papers [20,21]. Gas permeation experiments have been carried out in a home-made constant volume system that has been previously described elsewhere [21]. The membrane is placed inside a stainless steel permeation cell, which provides an effective membrane area of 15.55 cm^2^. The influence of temperature was studied in the range from 298 to 333 K using a water bath keeping the membrane module at isothermal conditions, feeding the single gases at 2–3 bar. Three single gas permeation experiments for each gas, temperature, and membrane material were considered to account for reproducibility assessment.

The gas permeability through the membrane is determined when steady state conditions are reached, according to Cussler [40] and as described in the previous works cited above. The permeability is the product of diffusivity (kinetic factor) and solubility (thermodynamic factor), known as the solution-diffusion model, Equation (1):

(1) P=D×S

The diffusion coefficient through the membranes is calculated from the transition regime of mass transfer through a dense material [41]:

(2) $$D=\delta^{2}/6\theta$$

where δ is the membrane thickness, and θ is the time lag taken by extrapolating, to the time axis, the linear part of the experimental curve in the accumulated volume of the permeate chamber *versus* the time plot. Estimated time lags range from 514 s to 2811 s for O_2_ and N_2_ permeation, respectively, for the membranes studied in this work.

The permeability and diffusivity is described in an Arrhenius form, as a function of temperature, by Equation (3) [42]:

(3) $$P=P_{0}\exp\left(-E_{P}/RT\right)\;\text{and}$$

(4) $$D=D_{0}\exp\left(-E_{D}/RT\right).$$

The solubility is described by the Van’t Hoff equation as follows:

(5) $$S=S_{0}\exp\left(-\Delta H_{S}/RT\right),$$

where *P*_0_, *D*_0_, and *S*_0_ are the pre-exponential factors for permeation, diffusion, and sorption, expressed in Barrer, cm^2^·s^−1^, and cm^3^(STP)/cm^3^, respectively, and *E_P_* and *E_D_* are the activation energy of permeation and diffusion, respectively, and Δ*H_S_* is the heat of sorption in kJ·mol^−1^.

The ideal selectivity is the intrinsic parameter that describes the ability of a membrane material to separate a certain pair of gases. It is usually defined as the ratio of the single gas permeability of the high to low permeating gas, which, for the O_2_/N_2_ gas pair aim of this study, is calculated by Equation (6) and as a contribution of solubility and diffusivity:

(6) $$\alpha \left(O_{2}/N_{2}\right)=\frac{P_{O_{2}}}{P_{N_{2}}}=\left(\frac{D_{O_{2}}}{D_{N_{2}}}\right)\left(\frac{S_{O_{2}}}{S_{N_{2}}}\right)=\alpha_{D}\times \alpha_{S}=\alpha_{P}.$$

## 4. Conclusions

In this work, the O_2_/N_2_ separation performance of mixed matrix membranes (MMMs) prepared from a highly permeable PTMSP polymer and small pore zeolites with Si/Al ratios of 1 and 5, and LTA, CHA, and Rho topologies was evaluated. Their oxygen and nitrogen permeation, diffusion, and sorption were determined at temperatures ranging from 298 to 333 K. The temperature influence has been well described in terms of Arrhenius and Van’t Hoff relationships, and the activation energies were thereby calculated. The introduction of the porous zeolite fillers in the membrane matrix increased the activation energy of permeation, leading to a higher influence of the temperature in the permeability than in the pristine polymer membranes. Moreover, the O_2_/N_2_ selectivity of the MMMs increased considerably compared with that of the pure polymer in the entire temperature range under study, probably because of the molecular sieving effect imparted by the introduction of the zeolites and the good interaction of the zeolites with the polymeric matrix. Although the permeability through the membrane decreased slightly, the permselectivity was improved because of the high increase in selectivity over the Robeson’s upper bound, by most of the MMMs at all temperatures.

These results highlight the good compatibility between these small-pore zeolites and the PTMSP in novel MMMs with improved permselectivity and the potential of these kinds of membranes to be used in oxygen-enriched air production processes, at higher temperatures than conventional separation processes, which could reduce the energy necessary to carry out the combustion process.

## Figures and Tables

**Table 1:** O_2_ permeabilities (Barrer) and O_2_/N_2_ permeability selectivities of the mixed-matrix membranes (MMMs) at different temperatures.

Membrane	*T* = 298 K		*T* = 313 K		*T* = 323 K		*T* = 333 K	
	*P* (O_2_)	α*_P_* (O_2_/N_2_)	*P* (O_2_)	α*_P_* (O_2_/N_2_)	*P* (O_2_)	α*_P_* (O_2_/N_2_)	*P* (O_2_)	α*_P_* (O_2_/N_2_)
PTMSP	10083 ± 936	1.32 ± 0.12	9072 ± 256	1.27 ± 0.04	8293 ± 851	1.27 ± 0.12	7024 ± 1050	0.94 ± 0.41
5 wt % CHA/PTMSP	885 ± 395	3.03 ± 0.96	1038 ± 270	2.79 ± 0.51	2965 ± 1321	7.25 ± 2.28	4308 ± 1608	8.43 ± 2.23
5 wt % LTA5/PTMSP	2727 ± 813	3.43 ± 0.72	3244 ± 193	3.97 ± 0.17	3642 ± 33	3.02 ± 0.02	4316 ± 1107	1.42 ± 2.23
5 wt % Rho/PTMSP	1368 ± 573	1.03 ± 0.63	2414 ± 921	1.99 ± 1.58	2707 ± 601	2.49 ± 1.66	5312 ± 1910	3.88 ± 1.02
20 wt % LTA1/PTMSP	2000 ± 198	4.91 ± 0.49	2051 ± 756	6.62 ± 2.44	2803 ± 1592	5.83 ± 3.31	4833 ± 2542	4.28 ± 2.25
5 wt % TMSP/PTMSP [23]	2346	1.92	2203	1.88	2018	1.82	–	–
LiA/PTMSP [17]	–	–	10390	1.45	–	–	–	–
20 vol % zeolite A/Matrimid	4.0	7.20	–	–	–	–	–	–
15 wt % zeolite NaA/PDMS [31]	330	2.23	403	2.12	–	–	493	1.65
50 wt % zeolite NaA/PDMS * [31]	221	2.23	284	2.12	–	–	330	1.82
20 wt % Zeolite A/PES [9]	–	–	0.35	6.0	–	–	–	–
15 wt % Zeolite A/PVAc [10]	–	–	0.45	7.45	–	–	–	–

* PDMS = polydimethylsiloxane.

**Table 2:** O_2_ diffusivities (10^8^·cm^2^·s^−1^) and O_2_/N_2_ diffusivity selectivities of the MMMs at different temperatures.

Membrane	*T* = 298 K		*T* = 313 K		*T* = 323 K		*T* = 333 K	
	*D* (O_2_)	α*_D_* (O_2_/N_2_)	*P*(O_2_)	α*_D_* (O_2_/N_2_)	*P* (O_2_)	α*_D_* (O_2_/N_2_)	*P* (O_2_)	α*_D_* (O_2_/N_2_)
PTMSP	5.47 ± 1.72	0.11 ± 0.04	3.50 ± 0.35	0.18 ± 0.03	3.46 ± 1.54	0.26 ± 0.09	3.40 ± 0.53	0.31 ± 0.06
5 wt % CHA/PTMSP	1.48 ± 0.28	1.83 ± 0.43	1.90 ± 0.07	1.84 ± 0.23	3.35 ± 0.95	2.86 ± 0.87	6.11 ± 1.87	4.30 ± 1.50
5 wt % LTA5/PTMSP	3.14 ± 0.82	1.84 ± 0.67	3.58 ± 0.57	1.79 ± 0.39	3.85 ± 0.11	0.98 ± 0.41	4.33 ± 1.02	1.04 ± 0.48
5 wt % Rho/PTMSP	1.17 ±0.13	0.37 ± 0.14	1.25 ± 0.14	0.51 ± 0.18	1.65 ± 0.22	0.74 ± 0.26	4.31 ± 1.15	1.02 ± 0.50
20 wt % LTA1/PTMSP	2.78 ± 1.01	1.84 ± 1.03	3.00 ± 1.08	1.90 ± 1.04	4.08 ± 1.83	2.40 ± 1.40	7.45 ± 3.53	3.74 ± 2.12

**Table 3:** O_2_ solubilities (cm^3^·(STP)·cm^−3^·cm·Hg^−1^) and O_2_/N_2_ solubility selectivities of the MMMs at different temperatures.

Membrane	*T* = 298 K		*T* = 313 K		*T* = 323 K		*T* = 333 K	
	*S*(O_2_)	α*_S_* (O_2_/N_2_)	*S*(O_2_)	α*_S_* (O_2_/N_2_)	*S*(O_2_)	α*_S_* (O_2_/N_2_)	*S*(O_2_)	α*_S_* (O_2_/N_2_)
PTMSP	18.43 ± 1.21	11.59 ± 3.22	25.92 ± 0.52	7.18 ± 1.02	23.97 ± 1.77	4.49 ± 0.55	20.66 ± 6.23	3.05 ± 0.72
5 wt % CHA/PTMSP	5.44 ± 1.14	0.76 ± 0.16	5.83 ± 1.12	0.79 ± 0.15	8.76 ± 0.43	1.25 ± 0.09	7.15 ± 0.46	1.00 ± 0.11
5 wt % LTA5/PTMSP	8.82 ± 0.68	2.08 ± 0.33	9.24 ± 1.54	2.10 ± 1.25	9.48 ± 0.31	1.58 ± 0.18	10.11 ± 0.80	1.46 ±0.77
5 wt % Rho/PTMSP	7.55 ± 0.58	1.87 ± 0.16	10.09 ± 0.54	1.89 ± 0.80	10.46 ± 4.89	2.05 ± 1.00	12.93 ± 0.44	3.83 ± 0.58
20 wt % LTA1/PTMSP	9.26 ± 5.71	6.40 ± 3.25	7.34 ± 1.06	4.16 ± 1.00	7.51 ± 1.87	4.95 ± 2.45	7.64 ± 2.42	2.43 ± 0.62

**Table 4:** Activation energies of permeation, diffusion, and sorption for the poly(1-trimethylsilyl-1-propyne) (PTMSP)-based MMM.

Membrane	*E_P_* (kJ/mol)		*E_D_* (kJ/mol)		−Δ*H_S_* (kJ/mol)	
	O_2_	N_2_	O_2_	N_2_	O_2_	N_2_
PTMSP	−8.19 ± 1.72	−12.60 ± 4.10	−11.02 ± 1.71	−17.29 ± 8.34	−2.34 ±1.89	5.36 ± 2.32
5 wt % CHA/PTMSP	39.9 ± 13.44	12.13 ± 3.17 ^1^	33.2 ± 13.16	12.85 ± 2.28 ^2^	6.51 ± 3.28	−1.02 ± 8.56
5 wt % LTA5/PTMSP	10.58 ± 4.27	42.29 ± 13.05 ^1^	7.37 ± 3.29	28.25 ± 1.65 ^2^	3.07 ± 1.56	12.21 ± 1.21
5 wt % Rho/PTMSP	29.65 ± 9.59	−5.04 ± 0.69 ^1^	27.82 ± 1.53	−12.35 ± 8.15 ^2^	11.95 ± 1.72	8.11 ± 1.76
20 wt % LTA1/PTMSP	19.78 ± 7.87	19.96 ± 10.7 ^2^	21.93 ± 5.24	8.54 ± 5.18	1.72 ± 1.00	16.50 ± 9.08

^1^ [21]; ^2^[20].
